# Exploring the Association of Hemoglobin Level and Adverse Events in Children with Cancer Presenting with Fever in Neutropenia

**DOI:** 10.1371/journal.pone.0101696

**Published:** 2014-07-14

**Authors:** Roland A. Ammann, Felix K. Niggli, Kurt Leibundgut, Oliver Teuffel, Nicole Bodmer

**Affiliations:** 1 Department of Pediatrics, University of Bern, Bern, Switzerland; 2 Division of Oncology, Department of Pediatrics, University of Zurich, Zurich, Switzerland; 3 Division of Oncology, Medical Services of the Statutory Health Insurance Baden-Württemberg, Tübingen, Germany; National Cancer Institute, United States of America

## Abstract

**Background:**

In children and adolescents with fever in neutropenia (FN) during chemotherapy for cancer, hemoglobin ≥90 g/L at presentation with FN had been associated with adverse events (AE). This analysis explored three hypothetical pathophysiological mechanisms potentially explaining this counterintuitive finding, and further analyzed the statistical association between hemoglobin and AE.

**Methods:**

Two of 8 centers, reporting on 311 of 421 FN episodes in 138 of 215 patients participated in this retrospective analysis based on prospectively collected data from three databases (SPOG 2003 FN, transfusion and hematology laboratories). Associations with AE were analyzed using mixed logistic regression.

**Results:**

Hemoglobin was ≥90 g/L in 141 (45%) of 311 FN episodes, specifically in 59/103 (57%) episodes with AE, and in 82/208 (39%) without (OR, 2.3; 99%CI, 1.1–4.9; *P = *0.004). In FN with AE, hemoglobin was bimodally distributed with a dip around 85 g/L. There were no significant interactions for center, age and sex. In multivariate mixed logistic regression, AE was significantly and independently associated with leukopenia (leukocytes <0.3 G/L; OR, 3.3; 99%CI, 1.1–99; *P* = 0.004), dehydration (hemoglobin_Presentation_/hemoglobin_8–72 hours_ ≥1.10 in untransfused patients; OR, 3.5; 99%CI, 1.1–11.4; *P* = 0.006) and non-moderate anemia (difference from 85 g/L; 1.6 per 10 g/L; 1.0–2.6; *P* = 0.005), but not with recent transfusion of packed red blood cells (pRBC), very recent transfusion of pRBC or platelets, or with hemoglobin ≥90 g/L as such.

**Conclusions:**

Non-moderate anemia and dehydration were significantly and relevantly associated with the risk of AE in children with cancer and FN. These results need validation in prospective cohorts before clinical implementation.

## Introduction

Fever in neutropenia (FN) is the most frequent potentially lethal complication of chemotherapy in pediatric patients with cancer [1]. The prospective multicenter SPOG 2003 FN study had investigated FN in children and adolescents with cancer. In its risk prediction analyses, mild/no anemia at presentation with FN, defined as hemoglobin level ≥90 g/L, was surprisingly found to be associated with adverse events (AE). Specifically, a hemoglobin level at presentation with FN (furthermore called hemoglobin) ≥90 g/L significantly and independently predicted AE in general, as well as the AE subgroups bacteremia, and serious medical complications [2]–[4].

This finding seems to contradict the well-known association of indicators of myelosuppression with the risk of AE. Besides, in the large literature on risk prediction in pediatric FN [5], hemoglobin had been described as a risk factor only twice, both in single-center retrospective studies, and with conflicting results. In Switzerland, high hemoglobin (>70 g/L) independently predicted severe bacterial infection [6], while in Brazil, low hemoglobin (<70 g/L) independently predicted severe infectious complications [7].

This additional analysis of SPOG 2003 FN data aimed to explore five hypotheses, three of them on pathophysiological and two on statistical mechanisms potentially underlying the association of hemoglobin level with AE in FN. The pathophysiological mechanisms explored were (1) suppressed erythropoiesis, hemolysis, or blood loss having led to transfusion of packed red blood cells (pRBC); (2) febrile transfusion reaction despite screening and the use of leukocyte depletion filters [8],[9]; and (3) dehydration or hemoconcentration [10],[11]. The statistical mechanisms explored were (4), non-optimal categorization of hemoglobin, which may be non-monotonously associated with AE, by dichotomization [12]; and (5) a direct effect of high hemoglobin on the risk of AE.

## Patients and Methods

### Patients, and management of fever in neutropenia

This retrospective analysis was essentially based on data from the prospective multicenter study SPOG 2003 FN, initiated by the Swiss Pediatric Oncology Group. This study had been open for patient recruitment by pediatric oncology centers in Switzerland and Germany from 2004 to 2007.

Details on eligible patients, and on management of FN have been published [2]–[4]. Patients with cancer aged 1 to 18 years at presentation with FN after non-myeloablative chemotherapy had been eligible. Multiple episodes of FN per patient had been allowed. Fever had been defined as axillary temperature either ≥38.5°C once, or ≥38.0°C during ≥2 hours [1]. Neutropenia had been defined as an absolute neutrophil count ≤0.5 G/L [1].

Hemoglobin had been determined as part of the routine blood cell count at presentation with FN, together with blood cultures and a physical examination. Patients had been hospitalized and treated with empirical intravenous broad spectrum antimicrobial therapy. The treating physician had decided on all further diagnostic and therapeutic measures including transfusions and follow-up determinations of hemoglobin. By institutional standard procedures, all pRBC and single-donor apheresis platelets had been leukocyte-depleted [8]. Few patients fulfilling restrictive predefined low-risk criteria at reassessment after 8 to 22 hours of inpatient-therapy and had been randomized in a low-risk subgroup study comparing response and safety rates of oral ciprofloxacin and amoxicillin administered in an outpatient setting versus continued standard treatment [13].

### Ethics statement

Patients, if able to judge, and their legal guardians had given written informed consent prior to study entry. The study had been conducted in accordance with the Declaration of Helsinki and the Guidelines on Good Clinical Practice. The protocol had been approved by the respective local and national ethics committees, and registered at www.clinicaltrials.com (NCT00107081), before starting patient accrual [2].

This retrospective analysis was approved by the Institutional Review Board of the Inselspital Bern (registration number 13–06–11), including waiver of renewal of informed consent. After matching, information was anonymized for analysis.

### Data bases used for this analysis

Only two of the eight SPOG 2003 FN study centers were willing to perform the additional workload of data collection required for participation in this additional retrospective analysis. Specifically, this data collection required extraction of data from the database of the transfusion facilities for data on transfusion before and after presentation with FN, and from the database of the hematology laboratories for results on follow-up hemoglobin determinations. The prospectively collected data from these two databases was added to the SPOG 2003 FN database after matching by initials, date of birth, and date of FN. Matching was performed centrally, and was always found to be unambiguous.

### Definition of adverse events

Details on AE definition have been published [2]–[4]. AE included serious medical complications due to infection [14]–[16], microbiologically defined infection, and radiologically confirmed pneumonia. Serious medical complications included death, complication requiring intensive care unit treatment, and potentially life-threatening complication as judged by the treating physician. Microbiologically defined infections included positive bacterial or fungal culture from a normally sterile body fluid or compartment, and detection of a viral antigen or product of polymerase chain reaction by a validated microbiological method. Adverse events were reported until the day when antimicrobial therapy had been stopped for 7 days, and severe neutropenia had resolved, whichever occurred later.

### Measures and definitions specific to the five hypotheses

A surrogate marker was defined for each of the three pathophysiological mechanisms explored. The surrogate marker for hypothesis (1), suppressed erythropoiesis, hemolysis, or blood loss having led to transfusion of pRBC, was recent transfusion of pRBC within 168 hours (7 days) before presentation with FN. The surrogate marker for hypothesis (2), febrile transfusion reaction, was very recent transfusion of pRBC, or single-donor apheresis platelets, within 24 hours before presentation with FN. The surrogate marker for hypothesis (3), dehydration or hemoconcentration, was hemoglobin ratio (Hb-ratio), estimated by the ratio of hemoglobin measured at presentation with FN, divided by hemoglobin measured at the first follow-up blood count, if this was performed 8 to 72 hours after presentation and only in patients not transfused with pRBC until then).

For the two statistical mechanisms explored, (4), non-optimal categorization of hemoglobin by dichotomization [12]; and (5) a direct effect of high hemoglobin on the risk of AE, no specific definitions were required.

### Statistics

Median, range and interquartile range (IQR) were calculated for non-normally distributed variables. Exact Fisher's and Fisher-Freeman-Halton tests, t-tests, and recursive partitioning by tree analysis [17] were used where applicable [12]. Smoothing by 2^nd^ to 8^th^ (deliberate overfitting) order linear regression on AE was used for figures.

Univariate and multivariate mixed logistic regression, with a random intercept per patient, was used for analysis of associations with the binary outcomes, because multiple FN episodes per patient were allowed [18]. In addition to known risk factors for AE, and hypothesis-specific characteristics, interaction terms of high hemoglobin (≥90 g/L) with recent transfusion of pRBCs, and with very recent transfusion of pRBCs/platelets, respectively, were analyzed. Odds ratio (OR), its 99% confidence interval (CI), and *P*-value were reported. For multivariate analysis, the backward variable selection procedure was used (*P*_in_ = 0.010), starting with the 4 respective outcome-specific characteristics known from SPOG 2003 FN, plus one characteristic each for the five hypotheses [12].

Two-sided tests were used throughout. Accounting for the multiple statistical tests performed, only *P*-values <0.01 were considered significant. Correspondingly, 99% CI were calculated. All exact analyses were performed using StatXact 10.0 (Cytel Software Corp., Cambridge, MA, USA), and all remaining analyses using R 2.15.1 (R Foundation for Statistical Computing, Vienna, Austria). Specifically, for mixed logistic regression the *sabre* procedure from the *sabreR* library was used [19].

## Results

### Patients and episodes of FN

Two of originally 8 SPOG 2003 FN centers participated in this analysis, in which thus 311 (74%) of 421 FN episodes occurring in 138 (67%) of 205 patients were included. The 110 episodes from the six centers not participating in this additional analysis had to be excluded. An AE was reported in 103 (33%) of these 311 FN episodes.

Sex, age at first FN, diagnostic group, and relapse status were not associated with inclusion versus exclusion (Table 1). The proportion of FN episodes with AE, however, was significantly higher in the episodes included than in those excluded, while the proportion of episodes with hemoglobin ≥90 g/L were comparable. (Table 1).

**Table 1:** Characteristics of Patients and of FN Episodes Excluded and Included into the Analysis.

	Patients and FN episodes			
Characteristic	SPOG 2003 FN	Excluded^a^	Included^a^	*P* ^b^
Patients	205	67 (33%)	138 (67%)	
Sex				0.37
Female	90	26 (29%)	64 (71%)	
Male	115	41 (36%)	74 (64%)	
Age at first FN				0.68
<4 years	55	16 (29%)	39 (71%)	
≥4 and <8 years	57	22 (39%)	35 (61%)	
≥8 and <12 years	45	15 (33%)	30 (67%)	
≥12 years	48	14 (29%)	34 (71%)	
Diagnostic group				0.25
Acute lymphoblastic leukemia	90	24 (27%)	66 (73%)	
Acute myeloid leukemia	23	6 (26%)	17 (74%)	
Lymphoma	16	5 (31%)	11 (69%)	
Tumor of CNS	22	8 (36%)	14 (64%)	
Solid tumor outside CNS	54	24 (44%)	30 (56%)	
Relapse status				0.66
Non-relapsed cancer	180	60 (33%)	120 (67%)	
Relapsed cancer	25	7 (28%)	18 (72%)	
Episodes of FN	421	110 (26%)	311 (74%)	
Episodes with adverse event	121	18 (15%)	103 (85%)	<0.001
Episodes with hemoglobin ≥90 g/L	189	48 (25%)	141 (75%)	0.82

FN, fever in neutropenia. ^a^Proportions of all patients/episodes in parentheses; ^b^ *P*-values from exact Fisher's and Fisher-Freeman-Halton tests regarding excluded versus included episodes or patients.

### Hemoglobin and adverse events

**Figure 1:**
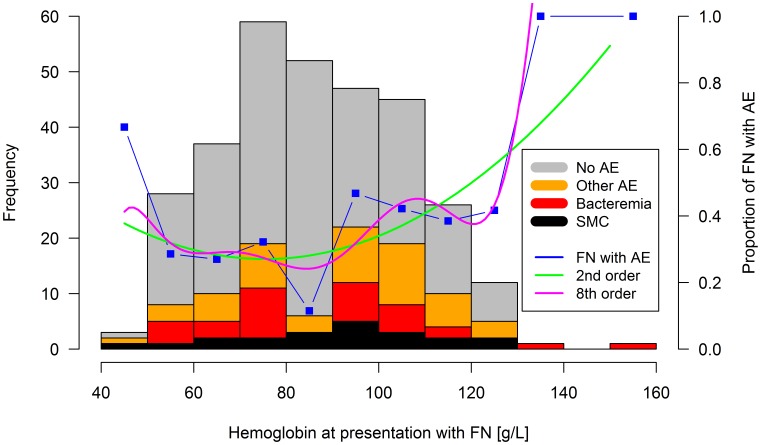
Adverse events versus hemoglobin at presentation. Frequency histogram of episodes of fever in neutropenia (FN; left vertical axis). Proportions of FN episodes with any adverse event (AE) measured, and by 2^nd^ and 8^th^ order regression smoothing (right vertical axis). SMC, serious medical complication.

The median hemoglobin at presentation with FN was 86 g/L (range, 44 to 151; IQR, 72 to 102). Hemoglobin was ≥90 g/L in 141 (45%) of the 311 FN episodes, specifically in 59 (57%) of 103 episodes with AE versus 82 (39%) of 208 without (OR, 2.3; 99% CI, 1.1 to 4.9; p = 0.004). Hemoglobin at presentation was bimodally distributed in FN with AE, with a dip around 85 g/L (moderate anemia), between two peaks at severe and at mild/no anemia. Correspondingly, the distribution of the proportions of FN with AE versus hemoglobin was U-shaped (Figure 1).

Center was not a significant confounder for the association of hemoglobin ≥90 g/L with AE (OR of interaction, 3.5; 99% CI, 0.7 to 19; p = 0.052). The same was true for sex (OR of interaction, 1.5, 99% CI, 0.4 to 6.5, p = 0.45) and age (four categories; all p-values for interaction >0.01) Based on these results, all further calculations were performed without stratification for center, sex, and age.

### Characteristics from SPOG 2003 FN

In univariate analysis, three of the four characteristics independently associated with AE in SPOG 2003 FN were as well significantly associated with AE in this analysis (hemoglobin ≥90 g/L, leukocyte count <0.3 G/L, platelet count <50 G/L), while the fourth (chemotherapy more intensive than ALL maintenance) was not.

### Three pathophysiological mechanisms explored

Hypothesis (1), suppressed erythropoiesis, hemolysis, or blood loss having led to transfusion of pRBC: A recent transfusion of pRBC, i.e., within 7 days before presentation with FN, had been reported in 97 (31%) of 311 FN episodes, 36 of them with AE, and 61 without. The mean hemoglobin was 91 g/L in these 97 episodes versus 85 g/L in the remaining 214 episodes (mean difference, 6.0 g/L; 99% CI, −0.5 to 12.5 g/L; p = 0.017). Recent transfusion of pRBC, and its interaction with hemoglobin ≥90 g/L, was not significantly associated with AE (Table 2).

**Table 2:** Characteristics Associated with Adverse Events During Fever in Neutropenia.

	Characteristic present in episodes		Univariate analysis		Multivariate analysis^a^	
Characteristics	with AE	without AE	OR (99%CI)	*P*	OR (99%CI)	*P*
Independently associated in SPOG 2003 FN						
Hemoglobin ≥90 g/L	59 of 103 (57%)	82 of 208 (39%)	2.3 (1.1 to 4.9)	0.004	Not significant	*-*
Chemotherapy more intensive than ALL maintenance	90 of 103 (87%)	179 of 208 (86%)	1.2 (0.4 to 3.6)	0.61	Not significant	*-*
Leukocyte count <0.3 G/L	54 of 103 (52%)	73 of 208 (35%)	2.3 (1.1 to 4.8)	0.004	3.3 (1.1 to 9.9)	*0.004*
Platelet count <50 G/L	69 of 103 (67%)	109 of 208 (52%)	1.9 (0.9 to 4.0)	0.018	Not significant	*-*
Hypotheses tested in this analysis						
H.2: Recent pRBC transfusion^b^	36 of 103 (35%)	61 of 208 (29%)	1.3 (0.6 to 2.7)	0.40	Not significant	-
Interaction with high hemoglobin	NA	NA	0.8 (0.2 to 3.7)	0.73	Not tested	*-*
H.3: Very recent pRBC/platelets transfusion^c^	16 of 103 (16%)	35 of 208 (17%)	0.9 (0.3 to 2.2)	0.71	Not significant	*-*
Interaction with high hemoglobin	NA	NA	0.8 (0.1 to 5.5)	0.75	Not tested	-
H.4: Dehydration (Hb-Ratio ≥1.10)^a^	57 of 74 (77%)	84 of 145 (58%)	3.1 (1.0 to 9.1)	0.007	3.5 (1.1 to 11.4)	0.006
Hb-Ratio as such (per 10%)^a^	NA	NA	1.4 (1.0 to 1.9)	0.020	Not tested	-
H.5: Non-moderate anemia (|85 - Hb|; per 10 g/L)	NA	NA	1.4 (1.0 to 1.9)	0.008	1.6 (1.0 to 2.6)	0.005
H.6: High hemoglobin as such (per 10 g/L)	NA	NA	1.2 (1.0 to 1.4)	0.024	Not significant	-

AE, adverse event; FN, fever in neutropenia; Hb, hemoglobin; NA, not applicable; pRBC, packed red blood cells. ^a^n = 219 instead of 311; ^b^within 7 days; ^c^within 24 hours.

Hypothesis (2), febrile transfusion reaction: A very recent transfusion of pRBC, i.e., within 24 hours before presentation with FN, had been reported in 34 (11%) of 311 FN episodes, and a transfusion of platelets in 39 (13%). This resulted in a total of 51 (16%) FN episodes with a very recent transfusion of pRBC and/or platelets, 16 of them with AE, and 35 without. Very recent transfusion of pRBC and/or platelets, and its interaction with hemoglobin ≥90 g/L, were not significantly associated with AE (Table 2).

**Figure 2:**
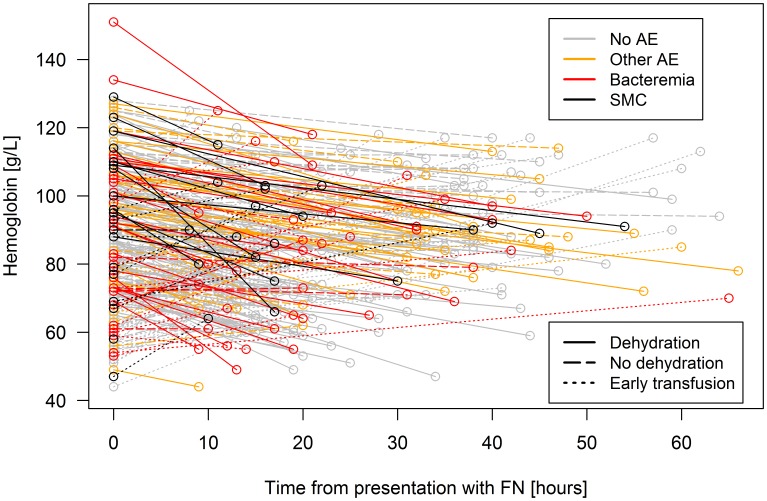
Course of hemoglobin used to calculate Hb-ratio. Hemoglobin at presentation, and within 72; AE, adverse event; SMC, serious medical complication.

**Figure 3:**
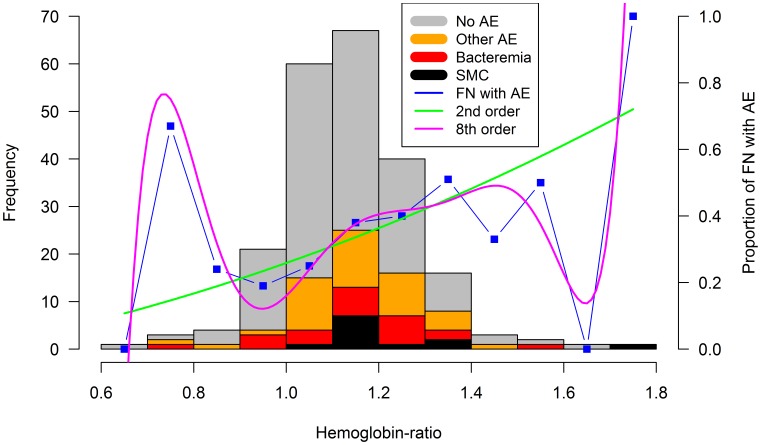
Adverse events versus dehydration, estimated by Hb-ratio. Frequency histogram of FN episodes (left vertical axis). Proportions of FN episodes with any AE measured, and by 2^nd^ and 8^th^ order regression smoothing (right vertical axis).

Hypothesis (3), dehydration or hemoconcentration: Within 8 to 72 hours after presentation with FN, a follow-up hemoglobin was determined in 297 of 311 episodes (95%; median time, 20 hours; range, 8 to 66, IQR, 14 to 35). In 78 (25%) of these episodes, however, transfusion with pRBC had been started before determination of follow-up hemoglobin. A Hb-ratio could thus be calculated in 219 (70%) FN episodes (Figure 2). The median Hb-ratio was 1.13 (range, 0.66 to 1.73; IQR, 1.07 to 1.24; Figure 3). Recursive partitioning indicated 1.09 as significant limit to discriminate between episodes with versus without AE. For clinical convenience, the limit of Hb-ratio defining dehydration was set at ≥1.10. Dehydration was present in 141 (64%) of 219 episodes. There was a significant, essentially linear, and thus monotonous association of Hb-ratio with AE (Table 2, Figure 3).

### Two statistical mechanisms explored

**Figure 4:**
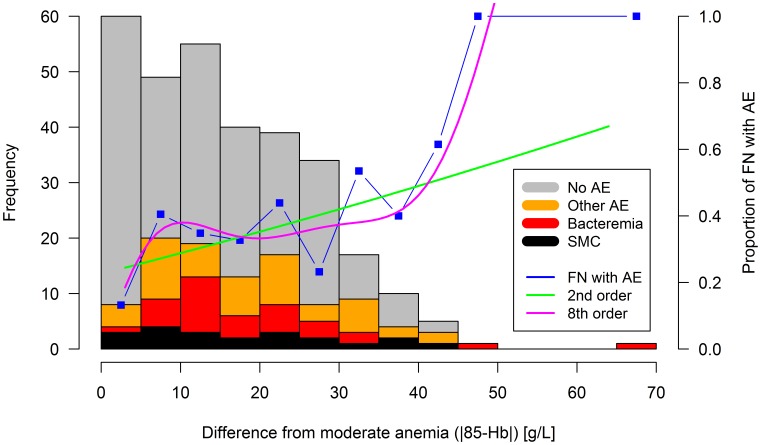
Adverse events versus the difference from moderate anemia. Frequency histogram of FN episodes (left vertical axis). Proportions of FN episodes with any AE measured, and by 2^nd^ and 8^th^ order regression smoothing (right vertical axis).

Hypothesis (4), non-optimal categorization of hemoglobin by dichotomization: Recursive partitioning of the association of hemoglobin at presentation with AE was recalculated in these 311 FN episodes. It revealed a primary split of the regression tree at ≥91 g/L, with a secondary split at ≥81 g/L. Moderate anemia was associated with the lowest risk (81 to 90 g/L; 12% with AE), while both severe anemia (<81 g/L, 31%) and mild/no anemia (≥91 g/L, 44%) were associated with a higher risk (Figure 1). This essentially U-shaped association between hemoglobin and the risk of AE was confirmed by 2^nd^ and higher order linear regression smoothing. The minimum risk estimated by 2^nd^ order linear regression smoothing was at 75 g/L. Only using 8^th^ or higher order linear regression smoothing, this minimum reached the range of 81 to 90 g/L (Figure 1). For clinical convenience, instead of using a complex categorization of hemoglobin, the U-shaped association for AE was integrated into a single variable, called non-moderate anemia, defined as the absolute value of the difference of hemoglobin from 85 g/L, |85-Hb|. There was a significant, essentially linear, and thus monotonous association of this measure of non-moderate anemia with AE (Figure 4, Table 2).

Hypothesis (5), direct effect of high hemoglobin on the risk of AE: High hemoglobin, analyzed as continuous instead of categorized variable, was not significantly associated with AE (Table 2).

### Multivariate analysis

Multivariate analysis was performed in the set of 219 FN episodes in which Hb-ratio as measure of dehydration and hemoconcentration was defined. Three characteristics were significantly and independently associated with AE (74 of 219 episodes; 34%). These were leukocyte count <0.3 G/L, dehydration defined as Hb-ratio ≥1.10, and non-moderate anemia defined as |85-Hb| (Table 2).

## Discussion

This analysis explored three pathophysiological and two statistical mechanisms potentially explaining the unexpected association of a hemoglobin level ≥90 g/L with an increased risk of AE during FN in pediatric patients with cancer, which had been found in SPOG 2003 FN. Two of these five hypothetical mechanisms were confirmed.

Of the three pathophysiological mechanisms, dehydration or hemoconcentration (hypothesis 3) was confirmed. It was estimated by a Hb-ratio ≥1.10, which was significantly and independently associated with AE. Hypotheses (1), suppressed erythropoiesis, hemolysis, or blood loss having led to transfusion of pRBC), and (2), febrile transfusion reaction, however, were falsified.

Of the two statistical mechanisms, non-optimal categorization of hemoglobin by dichotomization (hypothesis 4) was confirmed. A non-monotonous association of hemoglobin with the risk of AE was found, revealing that not only mild/no anemia, but as well severe anemia was associated with AE. This was integrated into the notion of non-moderate anemia, estimated here by |85-Hb| (absolute value of the difference of hemoglobin from 85 g/L). Hypothesis (5), a direct effect of high hemoglobin on the risk of AE, however, was falsified.

Dehydration as one of the main mechanisms confirmed here was assessed only indirectly by estimating the surrogate marker Hb-ratio. This relies on the follow-up determination of hemoglobin before start of any pRBC transfusion. Correspondingly, dehydration was not assessable in 30% of FN episodes. Interestingly, dehydration was associated with the risk of AE independently, i.e., in addition to, non-moderate anemia at presentation. The pathophysiological mechanism of this association, i.e., if dehydration is itself a cause of AE, or if it is an effect of other causes of AE, remains unanswered here.

The hypothesized non-monotonous association of hemoglobin with the risk of AE was confirmed here. It was found to be U-shaped, both by graphical (Figure 1) and test-based analysis (Table 2). Like all continuously measured variables in SPOG 2003 FN, hemoglobin at presentation had been dichotomized for use in the prediction models based on recursive partitioning, and respecting clinically useful limits [2]. The limit chosen, ≥90 g/L, had been exactly at the main tree split found for AE (higher risk of AE ≥90 g/L). A second significant split at ≥80 g/L (higher risk of AE <80 g/L) had deliberately not been used, because this would have led to three instead of two categories. Of course, this dichotomization reflects only the right-hand part of the U-shaped association found here, while the left-handed part essentially reflects myelosuppression. Correspondingly, the measure of non-moderate anemia, integrating both parts of the U, overruled both hemoglobin ≥90 g/L (right-handed part of U), and platelet count <50 G/L (left-handed part of U, myelosuppression) in multivariate analysis here. The fact that leukocyte count <0.3 G/L as a second marker of myelosuppression, remained in the multivariate model, underlines the strong association of myelosuppression with the risk of AE.

When compared to published results, the U-shaped association of hemoglobin and risk of AE found here is compatible with both associations published until today, which seem to contradict each other only at first sight. The association of high hemoglobin (>70 g/L) with severe bacterial infection found in Switzerland [6] corresponds to the right-handed part of the U, and the association of low hemoglobin (<70 g/L) with severe infectious complications found in Brazil [7] corresponds with its left-handed part, reflecting additionally different transfusion strategies. Furthermore, this U-shaped association seems to be robust, because the splits found by recursive partitioning here were nearly identical to those found in SPOG 2003 FN (primary split, ≥91 versus ≥90 g/L; secondary split, ≥81 versus ≥80 g/L) [2].

Besides the 5 clinical decision rules (CDR) mentioned above [2]–[4],[6],[7], only 3 of 13 further recently reviewed [5],[20] studies generating CDRs for pediatric FN reported non-significant univariate associations of hemoglobin with different AE [21]–[23]. Since hemoglobin is routinely assessed at presentation with FN, this may well represent underreporting of negative results. These in turn may be due to suboptimal techniques of analysis of non-monotonous associations by methods suited for the analysis of monotonous associations only.

For clinical use, the replacement of the current CDR based on the SPOG 2003 FN results [2],[3] by a modified rule including non-moderate anemia is not proposed for three reasons: First, the aim of this analysis was not to derive a new CDR, but to explore the mechanisms of a surprising aspect of the current CDR. Second, replacing a partially validated CDR by non-validated updates prevents full validation [24],[25]. Third, only 8^th^ order linear modeling confirmed the risk of AE being lowest in the region of 80 to 89 g/l hemoglobin, while 2^nd^ to 7^th^ order modeling located this dip in lower hemoglobin regions. This indicates that the proposed risk factor non-moderate anemia, |85-Hb|, may well result from overfitting, thus reducing generalizability to other patients [12].

Though this is an *a posteriori* analysis, it is entirely based on prospectively collected data in a multicenter setting, with reliable data on a large number of FN episodes in pediatric patients with a wide spectrum of malignancies, and a wide spectrum of AE detected. The wide spectrum of AE might be seen as a drawback, but serious AE are frequently detected late in the course of FN [4],[26], and sometimes are preceded by apparently non-serious AE [27]. Because of incomplete participation of former SPOG 2003 FN study centers, the proportions of FN episodes with AE, and with SMC, were significantly higher in the episodes included than in those excluded. This is due to the fact that both centers participating in this analysis had recruited patients and FN episodes without bias, while four of the remaining six centers had preferentially recruited patients with low risk of complications [4]. The validity of the results presented here is thus not hampered. By definition, Hb-ratio as surrogate marker of dehydration could not be calculated in all episodes. This reduced the number of episodes available for analyzing hypothesis 3, and for multivariate analysis, which may have led to false negative results. More commonly used surrogate markers for dehydration like changes in weight, urine specific gravity, or blood urea nitrogen were not used because they had not been systematically assessed prospectively. The use of mixed regression techniques prevented potential drawbacks as a result of the inclusion of multiple episodes of FN per patient [18].

In conclusion, hemoglobin at presentation with FN, specifically both severe and mild/no anemia versus moderate anemia, was associated significantly and relevantly with the risk of AE in children. This non-monotonous association requires categorization into at least three categories to become detectable. These results need validation in other prospective cohorts before clinical implementation, because results may be different in other patient populations. Dehydration was found to be independently associated with AE. In future studies, it should be assessed systematically and directly, instead of using a surrogate marker like Hb-ratio, in order to further elucidate its value in prediction of AE in FN.
